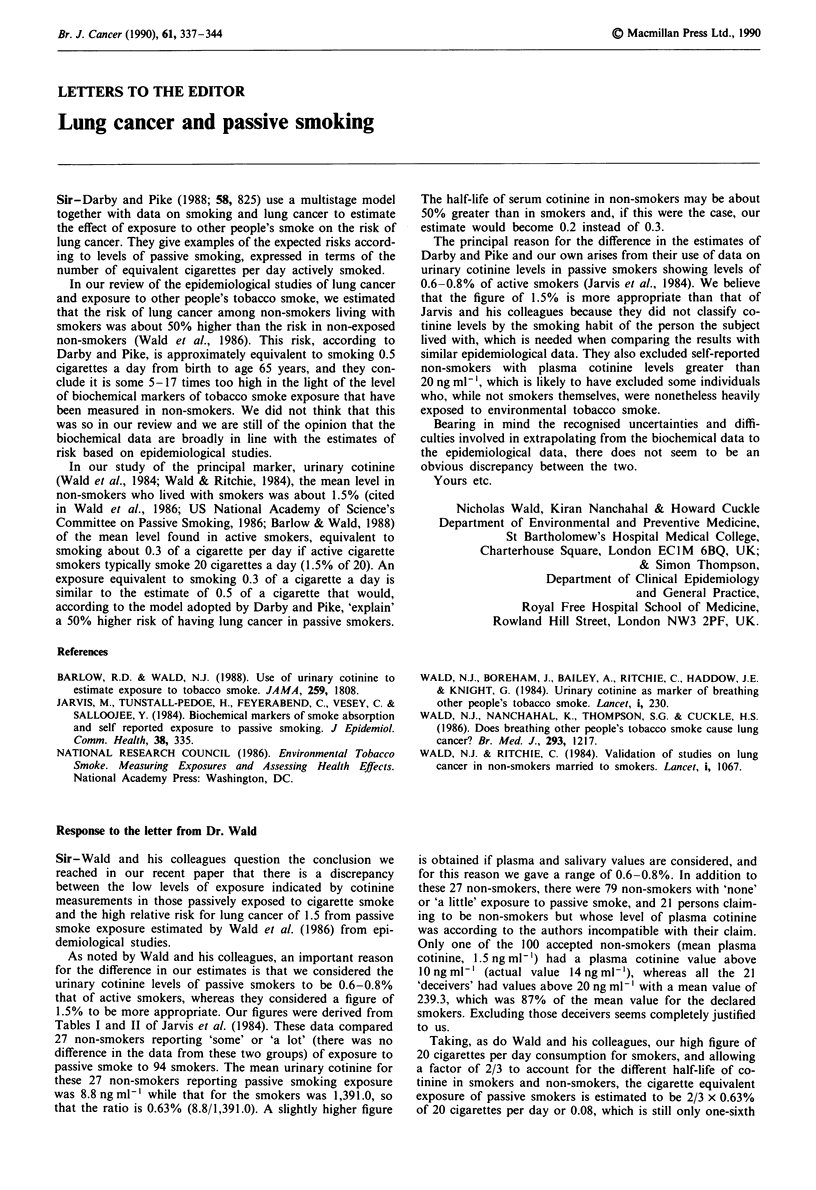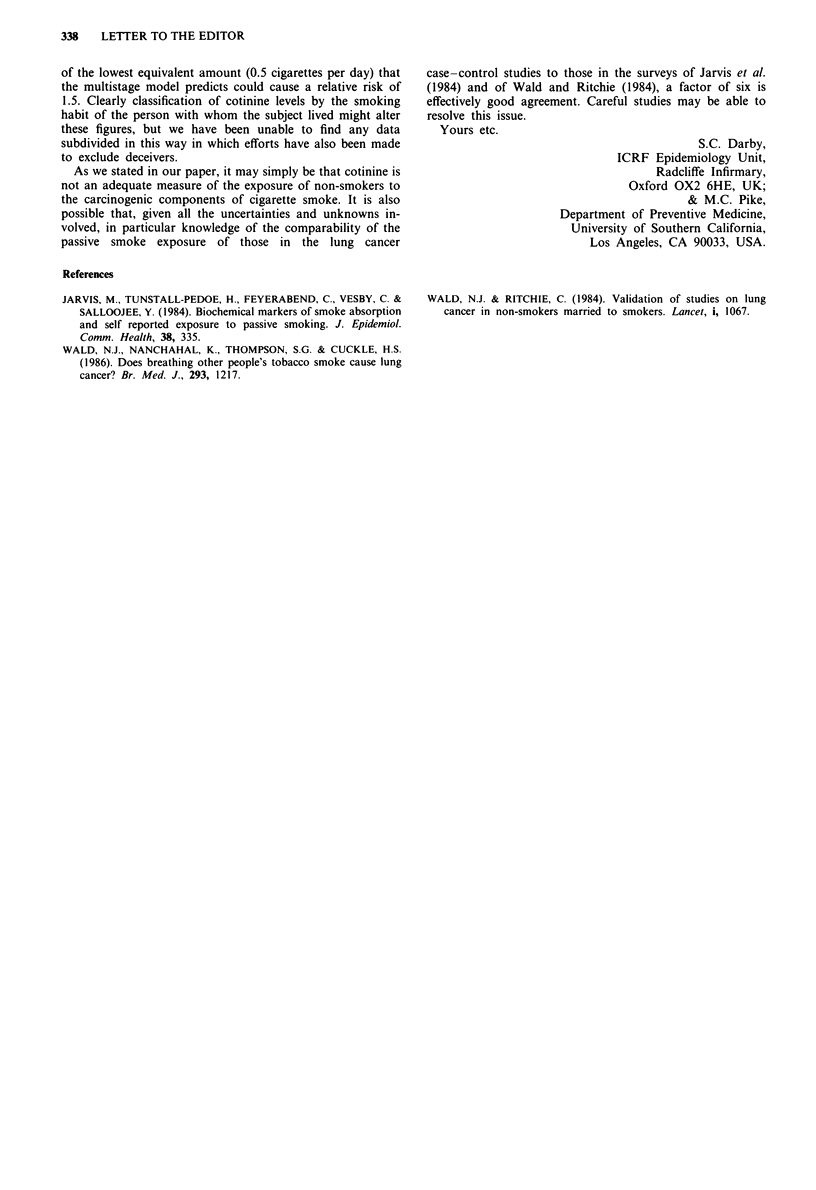# Response to letter from Wald et al

**Published:** 1990-02

**Authors:** S.C. Darby, M.C. Pike


WALD, N.J., NANCHAHAL, K., THOMPSON, S.G. & CUCKLE, H.S. (1986). Does breathing other people's tobacco smoke cause lung cancer? *Br. Med. J.*, **293**, 1217.

WALD, N.J. & RITCHIE, C. (1984). Validation of studies on lung cancer in non-smokers married to smokers. *Lancet*, **i**, 1067.

### Response to the letter from Dr. Wald

Sir–Wald and his colleagues question the conclusion we reached in our recent paper that there is a discrepancy between the low levels of exposure indicated by cotinine measurements in those passively exposed to cigarette smoke and the high relative risk for lung cancer of 1.5 from passive smoke exposure estimated by Wald *et al.* (1986) from epidemiological studies.

As noted by Wald and his colleagues, an important reason for the difference in our estimates is that we considered the urinary cotinine levels of passive smokers to be 0.6–0.8% that of active smokers, whereas they considered a figure of 1.5% to be more appropriate. Our figures were derived from Tables I and II of Jarvis *et al.* (1984). These data compared 27 non-smokers reporting 'some' or 'a lot' (there was no difference in the data from these two groups) of exposure to passive smoke to 94 smokers. The mean urinary cotinine for these 27 non-smokers reporting passive smoking exposure was 8.8 ng $ml^{-1}$ while that for the smokers was 1,391.0, so that the ratio is 0.63% (8.8/1,391.0). A slightly higher figure is obtained if plasma and salivary values are considered, and for this reason we gave a range of 0.6–0.8%. In addition to these 27 non-smokers, there were 79 non-smokers with 'none' or 'a little' exposure to passive smoke, and 21 persons claiming to be non-smokers but whose level of plasma cotinine was according to the authors incompatible with their claim. Only one of the 100 accepted non-smokers (mean plasma cotinine, 1.5 ng $ml^{-1}$) had a plasma cotinine value above 10 ng $ml^{-1}$ (actual value 14 ng $ml^{-1}$), whereas all the 21 'deceivers' had values above 20 ng $ml^{-1}$ with a mean value of 239.3, which was 87% of the mean value for the declared smokers. Excluding those deceivers seems completely justified to us.

Taking, as do Wald and his colleagues, our high figure of 20 cigarettes per day consumption for smokers, and allowing a factor of 2/3 to account for the different half-life of cotinine in smokers and non-smokers, the cigarette equivalent exposure of passive smokers is estimated to be $2/3 \times 0.63\%$ of 20 cigarettes per day or 0.08, which is still only one-sixth of the lowest equivalent amount (0.5 cigarettes per day) that the multistage model predicts could cause a relative risk of 1.5. Clearly classification of cotinine levels by the smoking habit of the person with whom the subject lived might alter these figures, but we have been unable to find any data subdivided in this way in which efforts have also been made to exclude deceivers.

As we stated in our paper, it may simply be that cotinine is not an adequate measure of the exposure of non-smokers to the carcinogenic components of cigarette smoke. It is also possible that, given all the uncertainties and unknowns involved, in particular knowledge of the comparability of the passive smoke exposure of those in the lung cancer case–control studies to those in the surveys of Jarvis *et al.* (1984) and of Wald and Ritchie (1984), a factor of six is effectively good agreement. Careful studies may be able to resolve this issue.

Yours etc.

S.C. Darby,
ICRF Epidemiology Unit,
Radcliffe Infirmary,
Oxford OX2 6HE, UK;
& M.C. Pike,
Department of Preventive Medicine,
University of Southern California,
Los Angeles, CA 90033, USA.

**References**

JARVIS, M., TUNSTALL-PEDOE, H., FEYERABEND, C., VESBY, C. & SALLOOJEE, Y. (1984). Biochemical markers of smoke absorption and self reported exposure to passive smoking. *J. Epidemiol. Comm. Health*, **38**, 335.